# Revisiting Janus kinases as molecular drug targets for rheumatic diseases

**DOI:** 10.3389/fmed.2026.1716179

**Published:** 2026-01-26

**Authors:** Sunghark Kwon

**Affiliations:** 1 Bright College, Hankyong National University, Anseong, Republic of Korea; 2 Gyeonggi Regional Research Center, Hankyong National University, Anseong, Republic of Korea

**Keywords:** autoimmune disorder, Janus kinase, kinase inhibitor, rheumatic disease, signal transducer and activator of transcription protein

## Abstract

Janus kinase (JAK) family members, as upstream regulators, phosphorylate not only themselves but also cytokine receptors and signal transducer and activator of transcription (STAT) proteins in the JAK-STAT signaling pathway. The JAK-STAT pathway is associated with various cellular processes, such as cell proliferation, cell death, and immune responses. Considering that the JAK-STAT pathway is involved in immunity, dysfunctional JAKs can cause autoimmune diseases, including rheumatoid arthritis. Therefore, several inhibitors have been developed to inhibit the function of JAKs in the case of abnormal JAK-STAT signaling. Emerging structural data on JAKs highlight the opportunities to design selective inhibitors that can overcome mutation-driven resistance. Therefore, novel JAK inhibitors need to be developed. In this review, we discuss the principal structural features of JAKs, focusing on the active site. In addition, we summarized the updated JAK inhibitors indicated for rheumatoid arthritis that are available in the pharmaceutical market. The binding modes of JAK inhibitors have also been described. Based on the structural analysis of JAKs and their inhibitors, we propose strategies for developing next-generation JAK inhibitors.

## 1 Introduction

The Janus kinase (JAK)-signal transducer and activator of transcription (STAT) pathway is a cell signaling pathway that has gained considerable attention in the fields of immunology and medicine. JAK-STAT signaling is associated with diverse cellular processes, such as cell division, cell death, tumor formation, and immune responses, including inflammatory responses (1–8). A group of proteins is involved in this signaling pathway. First, cytokines initiate the JAK-STAT signaling pathway by binding to dimeric transmembrane cytokine receptors, and as upstream regulators, JAKs bind to dimeric receptors and undergo autophosphorylation (9). Activated JAKs sequentially phosphorylate receptors and STATs to bind to the phosphorylated receptors, and STATs form a dimer and translocate to the nucleus (10). Finally, dimeric STATs bind to their target genes, resulting in transcription (10). Therefore, JAKs are upstream regulatory proteins in the JAK-STAT signaling pathway.

The JAK family comprises four members: JAK1, JAK2, JAK3, and tyrosine kinase2 (TYK2) (11, 12). All consist of four domains: (1) kinase (JH1), (2) pseudokinase (JH2), (3) Src homology 2 (SH2), and (4) band-4.1 protein, ezrin, radixin, and moesin (FERM) domains (13). However, in addition to sequence variations, the four JAK members exhibit different levels of expression depending on the cell type (13). Specifically, JAK1, JAK2, and TYK2 are ubiquitously produced in most cells, whereas JAK3 is primarily produced in hematopoietic and lymphoid cells (14). Although the four JAK members exhibit partial sequence variations, they share the stoichiometric properties of a dimeric functional unit (15, 16). JAKs can form homodimers or heterodimers, depending on their binding receptors (4, 17). The diversity of dimer formation indicates that specific cytokines and receptors recruit specific JAKs. Nonetheless, considering that most JAKs are associated with immune responses, it is reasonable to assume that a malfunctioning JAK in the upstream JAK-STAT pathway can cause anomalous immune responses, such as autoimmune responses.

To date, several JAK inhibitors targeting one or more JAK members have been developed and introduced in the global pharmaceutical market, including ruxolitinib (18–23), tofacitinib (24, 25), oclacitinib (26), baricitinib (27, 28), peficitinib (29–31), upadacitinib (32–34), fedratinib (23, 35, 36), delgocitinib (37), filgotinib (38), abrocotonib (39, 40), pacritinib (23, 41), deucravacitinib (42), ritlecitinib (43), momelotinib (44), golidocitinib (45), and deuruxolitinib (46). Each JAK inhibitor has specific targets and indications. JAK inhibitors target one or more members of the JAK family. Although these inhibitors show some target specificity, most bind to the ATP-binding site in the JH1 domain, and deucravacitinib binds to that in the JH2 domain to allosterically inhibit catalytic function (47–50). This suggests that the detailed architectures of the respective ATP-binding sites exhibit subtle structural diversity depending on the JAK type. Therefore, the development of novel JAK inhibitors requires generality for kinases and specificity for individual proteins.

The JH1 domain of the four JAK domains shows the structural dynamics of JAK activity. This domain contains several regions that exhibit conformational and locational diversity. Specifically, they correspond to the C-helix (αC), P-loop (or G-loop) in the N-terminal lobe, DFG motif, and A-loop in the C-terminal lobe, which are associated with the formation of ATP-binding sites (47). Notably, the A-loop exhibits two distinct conformations depending on ATP binding: active and inactive (51). The currently available JAK inhibitors bind to the ATP-binding site in the active form of the A-loop (47–50). However, this development strategy has an intrinsic limitation in terms of specificity because all kinases have their respective ATP-binding pockets, which eventually leads to unexpected side effects. Thus, current studies on the development of JAK inhibitors tend to focus on drug design based on possible binding pockets formed by the inactive A-loop (52, 53). Obtaining structural information on this conformation may enable the revolutionary design of novel JAK inhibitors with high specificity.

In this review, we summarize the latest JAK inhibitors introduced into the global pharmaceutical market. The structural features of JAKs are also discussed, focusing on the ATP-binding site and conformational changes in the A-loop. Based on the structural analysis of JAKs, we suggest strategies for developing novel JAK inhibitors with increased drug specificity. This review provides structural insights into the design of rational JAK inhibitors and presents a novel therapeutic paradigm for JAK-driven immune diseases, including rheumatic diseases.

## 2 JH1 domain structure of JAK2

**Figure 1:**
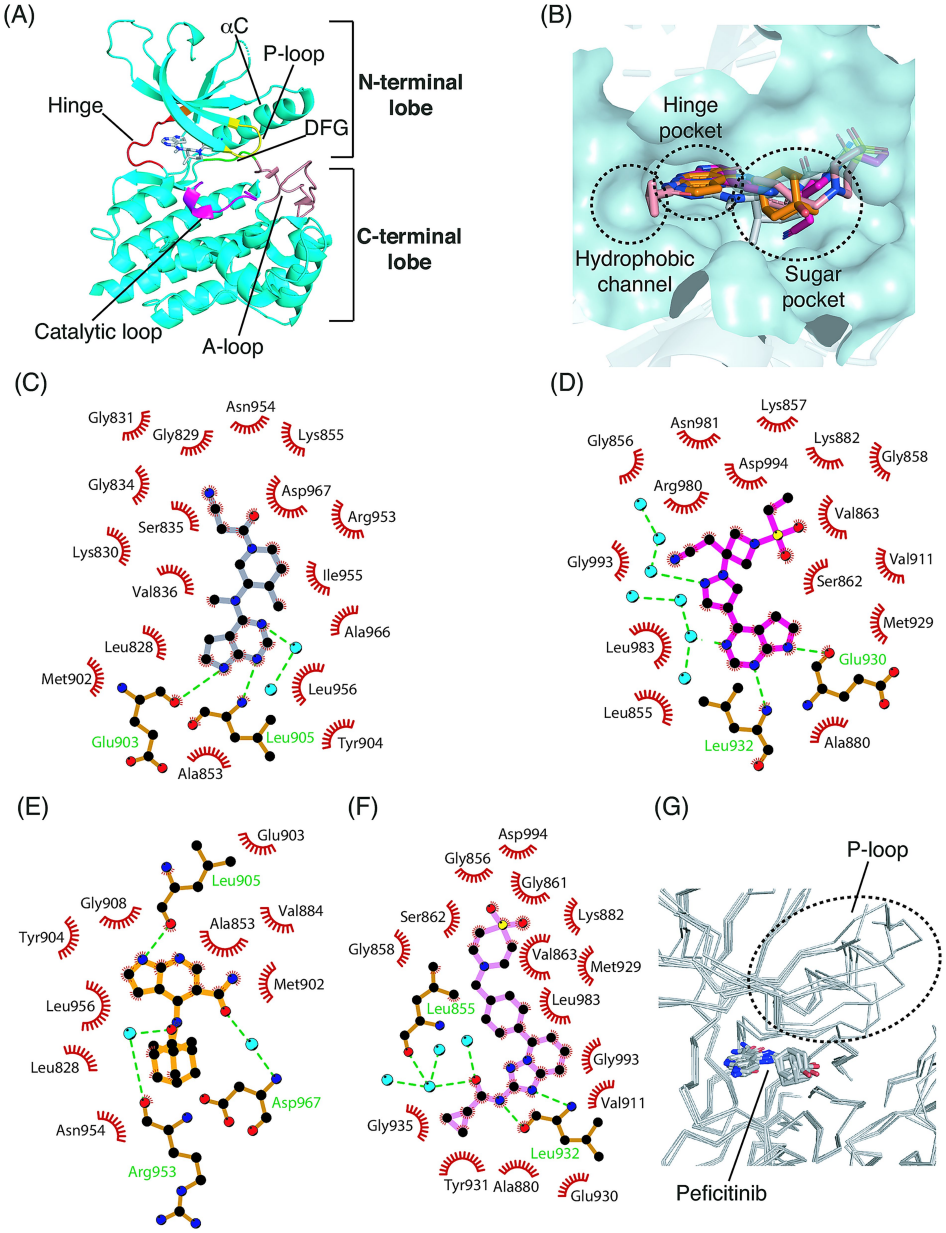
Structure of the JH1 domain of JAK2 and binding modes of JAK inhibitors. **(A)** Tertiary structure of the JH1 domain. The entire structure of JAK2 (PDB ID: 3EUP) is represented as cartoon. Tofacitinib is depicted as sticks. **(B)** Detailed pockets comprising the ATP-binding site. The hydrophobic back pocket is omitted for clarity. Tofacitinib (gray), baricitinib (magenta), peficitinib (orange), and filgotinib (salmon) are superimposed as sticks. Schematic diagrams of the binding modes of tofacitinib **(C)**, baricitinib **(D)**, peficitinib **(E)**, and filgotinib **(F)**, showing their interactions with adjacent residues. The hinge residues of JAK3 (JAK1/JAK2/TYK2) are Glu903 (Glu957/Glu930/Glu979), Tyr904 (Phe958/Tyr931/980), and Leu905 (Leu959/Leu932/Val981). Cyan circles and green dashed lines indicate water molecules and hydrogen bonds, respectively. **(G)** Superimposition of the four JAK members in complex with peficitinib.

Each JAK family member (JAK1-3 and TYK2) has four domains (JH1, JH2, SH2, and FERM) (13). However, from the perspective of JAK drug targeting, the JH1 domain is the most important component of JAKs because it plays an essential role in catalysis. In the JH1 domain, the N- and C-terminal lobes are connected via a hinge (Figure 1A). The JH2 domain is similar to the JH1 domain in terms of its amino acid sequence and tertiary structure (13). However, the JH2 domain exhibits lower catalytic activity than the JH1 domain (54–56). The JH2 domain is assumed to be an evolutionary product of *JH1* gene duplication. Herein, we briefly describe the structure of the JH1 domain of JAK2, as the crystal structures of JAK2 in complex with tofacitinib, baricitinib, peficitinib, and filgotinib have been determined.

The active site in the JH1 domain consists of the hinge region, P-loop, αC, catalytic loop, and A-loop (Figure 1A). The spatial architecture of the active site depends on the conformation of the A-loop. If the A-loop adopts an inactive conformation, the ATP molecule cannot bind to its active site. Therefore, the A loop must adopt an active conformation for ATP binding. The active site in the activated form provides four pockets for ATP binding: the hinge binder pocket, sugar pocket, hydrophobic channel, and hydrophobic back pocket (Figure 1B). This spatial configuration, which affects the specificity of JAKs, corresponds to the pharmacophore features of JAK inhibitors (56). Therefore, it is reasonable to assume that the shapes of high-affinity JAK inhibitors are complementary to these four pockets.

## 3 Binding modes of approved JAK inhibitors for rheumatoid arthritis

To date, 16 JAK inhibitors have been approved, mainly by the U. S. Food and Drug Administration (FDA), European Medicines Agency, and Japan, and have been launched in the pharmaceutical market. Some of these inhibitors are indicated for the treatment of rheumatoid arthritis. These include tofacitinib, baricitinib, peficitinib, upadacitinib, and filgotinib, all of which, except fedratinib, satisfy Lipinski’s rule of five. The inhibitor profiles are presented in Table 1.

**Table 1:** Profiles of representative JAK inhibitors.

Inhibitor	Trade name	Target	Domain	Approval year	Indications	Adverse effects	Mw (Da)	LogP	PDB ID
Ruxolitinib	Jakafi Jakavi Opzelura	JAK1 JAK2	JH1	2011 (US) 2012 (EU) 2014 (Japan)	- Myelofibrosis - Polycythemia vera - Graft- versus- host disease	- Thrombocytopenia - Anemia - Neutropenia	306	3.47	6VGL 6VNK 6WTN
Tofacitinib	Xeljanz Neojanz Jaquinus Tofacinix	JAK1 JAK3	JH1	2012 (US) 2013 (Japan) 2017 (EU)	- Rheumatoid arthritis - Psoriatic arthritis - Ankylosing spondylitis - Ulcerative colitis	- Upper respiratory tract infections - Headache - Diarrhea	312	0.84	3EYG 3FUP 3LXK 3LXN
Baricitinib	Olumiant	JAK1 JAK2	JH1	2017 (EU) 2017 (Japan) 2018 (US)	- Rheumatoid arthritis - Alopecia areata - COVID- 19	- Upper respiratory tract infections - Hypercholesterolemia	371	1.20	6VN8 6WTO
Peficitinib	Smyraf	JAK1 JAK3	JH1	2019 (Japan) 2020 (Korea)	- Rheumatoid arthritis	- Herpes zoster	303	2.86	6AAH 6AAJ 6AAK 6AAM
Upadacitinib	Rinvoq	JAK1	JH1	2019 (US) 2019 (Japan) 2019 (EU)	- Rheumatoid arthritis - Psoriatic arthritis - Ankylosing spondylitis - Ulcerative colitis - Atopic dermatitis	- Upper respiratory tract infections	380	2.91	–
Fedratinib	Inrebic	JAK2	JH1	2019 (US) 2021 (EU)	- Myelofibrosis	- Diarrhea - Nausea	524	5.90	6VNE
Delgocitinib	Corectim	pan-JAK	JH1	2020 (Japan)	- Atopic dermatitis	- Paresthesia			7C3N
Filgotinib	Jyseleca	JAK1	JH1	2020 (EU) 2020 (Japan)	- Rheumatoid arthritis - Ulcerative colitis	- Nausea - Diarrhea	425	3.06	4P7E 5UT5
Abrocitinib	Cibinqo	JAK1	JH1	2021 (Japan) 2021 (EU) 2022 (US)	- Atopic dermatitis	- Upper respiratory tract infections - Headache	323	2.34	6BBU 6BBV
Pacritinib	Vonjo	JAK2	JH1	2022 (US)	- Myelofibrosis	- Diarrhea - Nausea	472	4.96	8BPV
Deucravacit-inib	Sotyktu	TYK2	JH2	2022 (US)	- Plaque psoriasis	- Upper respiratory tract infections	425	1.70	-
Ritlecitinib	Litfulo	JAK3 TYK2	JH1	2023 (US) 2023 (EU) 2023 (Canada)	- Alopecia areata	- Headache - Diarrhea - Acne	285	1.94	-
Momelotinib	Ojjaara	JAK1 JAK2	JH1	2023 (US)	- Myelofibrosis	- Thrombocytopenia - Hemorrhage	414	2.98	8BXH

Several crystal structures of JAKs complexed with JAK inhibitors have been elucidated (47, 49, 50, 57–60). Most proteins in these structures are JH1 domains corresponding to the kinase domain, but not the entire JAK protein. Nevertheless, these structures provide valuable information on the binding modes of JAK inhibitors. In this section, we discuss the binding modes of JAK inhibitors indicated for rheumatoid arthritis based on the structural information deposited in the Protein Data Bank. However, the structure of JAKs in complex with upadacitinib has not yet been reported. Therefore, the binding mode of upadacitinib is not described herein.

Tofacitinib, a first-generation JAK inhibitor, primarily acts on JAK3 and JAK1, with lower inhibition of JAK2 and TYK2. The typical adverse effects of tofacitinib include upper respiratory tract infections, headache, and diarrhea (61). Thus, the FDA requires a boxed warning about injury and death that may be caused by such risks to be included on the label. The binding mode of tofacitinib to JAK3 is described based on its complex structure (PDB ID: 3LXK) (44) (Figure 1C). The pyrrolopyrimidine ring interacts with Glu903 and Leu905 in the hinge region of the JH1 domain. Tofacitinib interacts with many adjacent hydrophobic residues.

Baricitinib is a first-generation JAK inhibitor. The most common adverse effects of baricitinib are upper respiratory tract infections and hypercholesterolemia (62). Previous preclinical studies have demonstrated that baricitinib is contraindicated during pregnancy (62). The binding mode of baricitinib to JAK2 is described based on its complex structure (PDB ID: 6VN8) (46) (Figure 1D). Similar to tofacitinib, the pyrrolopyrimidine ring binds to Glu930 and Leu932 in the hinge region. A group of water molecules forms a network at the active site. Specifically, three water molecules are connected via hydrogen bonds, and one of the edges of the water molecules interacts with the N14 nitrogen atom via a hydrogen bond. In addition, one water molecule in another water group forms a hydrogen bond with the N26 nitrogen atom. Similar to tofacitinib, baricitinib interacts with many adjacent hydrophobic residues.

Peficitinib is a first-generation JAK inhibitor. The typical adverse effects of peficitinib are herpes zoster-related diseases, including varicella (63). The binding mode of peficitinib to JAK3 is described based on its complex structure (PDB ID: 6AAK) (54) (Figure 1E). However, unlike tofacitinib and baricitinib, the hinge binder interacts with only one hinge residue. In addition, two oxygen atoms in the amide and hydroxide groups linked to the adamantyl group interact with Arg953 and Asp967, respectively, mediated by water molecules. Although peficitinib interacts with adjacent hydrophobic residues, the number of residues is lower than those of tofacitinib and baricitinib.

Filgotinib is a second-generation JAK inhibitor. The most common adverse effects of filgotinib are upper respiratory tract infections, nausea, urinary tract infections, and dizziness (64). Filgotinib has not been approved for the treatment of rheumatoid arthritis by the FDA owing to safety issues. The binding mode of filgotinib to JAK2 is described based on its complex structure (PDB ID: 4P7E) (59) (Figure 1F). In contrast to the first-generation JAK inhibitors, the hinge binder in filgotinib binds only to Leu932. The amino-triazolopyridine moiety is associated with the binding of the hinge. In addition, a three-membered water group interacts with the Leu855 backbone. Similar to tofacitinib and baricitinib, filgotinib interacts with several adjacent hydrophobic residues.

Structural information on the four JAK members in complex with their respective JAK inhibitors is necessary to elucidate why each JAK inhibitor exhibits different binding affinities depending on the JAK members. However, for most JAK inhibitors, except for tofacitinib and peficitinib, the complex structures are limited to some of the JAK members. Although tofacitinib has various indications, including rheumatoid arthritis, peficitinib is only indicated for rheumatoid arthritis. Peficitinib has remarkable specificity for JAK3 with an IC_50_ value of 0.7 nM, whereas it shows IC_50_ values of 3.9 nM, 5.0 nM, and 4.88 nM for JAK1, JAK2, and TYK2, respectively (65). Superimposition of the four JAK members in complex with peficitinib revealed the conformational diversity of peficitinib and the P-loop (Figure 1G). Specifically, as shown in Figure 1E, the two oxygen atoms in the hydroxide and amide groups of peficitinib interact with Arg953 and Asp967 of JAK3, via two water molecules. Considering that peficitinib adopts different conformations for other JAKs, such as JAK1, JAK2, and TYK2, this unique binding mode may render peficitinib specific to JAK3.

## 4 Exploring strategies for the development of next-generation JAK inhibitors

As all kinases have at least one ATP-binding site, medicinal chemists have focused on ATP-binding sites as molecular drug targets. The overwhelming majority of currently available kinase inhibitors bind to ATP-binding sites. Accordingly, these inhibitors contain molecular moieties similar to ATP. However, inhibitors designed to target specific kinases can bind to other kinases. Such latent risk can cause diverse side effects at the individual level. Therefore, drug selectivity is an important factor in the development of kinase inhibitors. Here, we briefly address several strategies for increasing the selectivity of JAK inhibitors.

As mentioned in section 2, the ATP-binding site has four small pockets (56). The most significant pocket is the hinge binder pocket, where the hinge binder moiety of the JAK inhibitors binds. Specifically, the hinge binder forms approximately three hydrogen bonds with the peptide components of the hinge region. In JAK1 (JAK2/JAK3/TYK2), the hinge region comprises Glu957 (Glu930/Glu903/Glu979), Phe958 (Tyr931/Tyr904/Tyr980), and Leu959 (Leu932/Leu905/Val981). Although various hinge binders have been designed, they typically interact with the hinge regions. As shown in Section 3, tofacitinib and peficitinib interact with Glu903 and Leu905 in JAK3, whereas baricitinib and filgotinib interact with Leu932 in JAK2. Thus, the hinge binder moiety should be limited to defined skeletons when designing JAK inhibitors.

The hydrophobic back pocket is also an important component of JAK inhibitor binding. This region contains a gatekeeper that determines whether access to JAK inhibitors is allowed (56). The Met956, Met929, Met902, and Met978 residues are the gatekeepers of JAK1, JAK2, JAK3, and TYK2, respectively. As shown in Figure 1, the gatekeepers interact with the hydrophobic moieties of JAK inhibitors. Thus, gatekeepers should be considered as key factors in the design of JAK inhibitors. If the gatekeeper is mutated to a bulkier residue, access to JAK inhibitors may be obstructed. Consequently, such mutations lead to drug resistance to established JAK inhibitors. Although natural gatekeeper mutations have not yet been found in JAKs, they must be considered essential for novel JAK inhibitor designs if this type of resistance occurs.

Another noteworthy aspect is that the hydrophobic channel is positioned at the entrance of the ATP-binding site. Although this region is a well-known part of the ATP-binding site, most existing JAK inhibitors lack a moiety that binds to this hydrophobic channel. Considering that hydrophobic interactions affect the affinity of the inhibitors, it is ironic that this moiety has not been considered essential. Interestingly, filgotinib contains a cyclopropyl group that interacts with the hydrophobic channel. This is interesting because filgotinib has relatively high selectivity for JAK1. If this functional group contributes to high selectivity, the hydrophobic moiety may be a critical factor affecting the selectivity of JAK inhibitors.

JAK inhibitors developed at an early stage, such as ruxolitinib, tofacitinib, and baricitinib, are usually classified as first-generation JAK inhibitors. These compounds exhibit relatively poor selectivity and act on diverse JAK members. On the other hand, the second-generation JAK inhibitors include upacitinib, fedratinib, filgotinib, abrocitinib, pacricitib, deucravacitinib, and ritlecitinib. The first-generation JAK inhibitors have relatively simple structures based on hinge binders and sugar moieties. Therefore, they generally have low molecular weights and fewer hydrogen bond donors and acceptors, whereas second-generation JAK inhibitors tend to have high molecular weights and more hydrogen bond donors and acceptors (Table 1). These differences indicate that the latter fills the ATP-binding site with bulkier moieties and more hydrogen-bond networks. Fedratinib, filgotinib, and pacritinib bind to hydrophobic channels, whereas abrocitinib binds extensively to sugar pockets. Therefore, next-generation JAK inhibitors with specificity should be developed based on the space-filling concept of the ATP-binding site, which shows subtle structural differences among JAK members.

Recently, structure-guided discovery of JAK inhibitors has been actively pursued. Potent JAK1-selective inhibitors were designed and synthesized based on the JAK1 structure (66). The pyrrolopyrimidine derivatives, including the piperidinyl fragment, were obtained using a combinatorial method. In addition, several groups have attempted to discover novel JAK inhibitors using *in silico* screening techniques, such as ligand-based screening, pharmacophore modeling, molecular docking, and molecular dynamics simulations (67).

These three pockets mentioned above are components of the ATP-binding site, indicating that JAKs share this architecture with other kinases. Hence, other binding sites must be explored for the selective binding of JAK inhibitors. Allosteric sites may be good candidates to satisfy this condition. For example, in the crystal structure of MEK1 in complex with compound (PD318088) (PDB ID: 1S9J), the compound binds to an allosteric site adjacent to the ATP-binding site (68). Interestingly, in this crystal structure, ATP binds to the ATP-binding sites. JAK2 also has an allosteric site that is a potential site for novel JAK inhibitors. These allosteric inhibitors induce conformational changes, thereby inhibiting catalytic activity (68).

The most dynamic part of JAKs is the A-loop, similar to that of other kinases. The architecture of the inactive (DFG-out) conformation of the A-loop is completely different from that of the active conformation (56). The inactive conformations of the A-loop are shown in the crystal structures of several kinases, such as BCR-ABL in complex with imatinib (PDB ID: 1IEP) (69), nilotinib (PDB ID: 3CS9) (70), ponatinib (PDB ID: 3OXZ) (71), and p38 MAPK in complex with sorafenib (PDB ID: 3GCS) (72). These complex structures provide valuable information on unique binding pockets in the DFG-out conformation. These binding sites are promising candidates for increasing the specificity of JAK inhibitors. Unfortunately, the DFG-out conformations of JAKs are unknown, making it difficult to develop novel JAK inhibitors that target DFG-out conformations. However, if such conformations can be identified, it would be possible to design next-generation JAK inhibitors specific to each JAK member.

## 5 Discussion

Type I interleukin receptors binding IL-6, 11, 13, 31, and the interferon *γ* receptor binding INF-γ are associated with rheumatoid arthritis (73). Type I interleukin receptors recruit JAK1 and JAK2/TYK2 in the JAK-STAT signaling pathway (73). The interferon γ receptor also binds JAK1 and JAK2 as a binary combination (73). Accordingly, the cytokine receptors related to rheumatoid arthritis share a common JAK combination of JAK1 and JAK2. This indicates that JAK 1 and JAK2 are inhibitor targets for treatment of rheumatoid arthritis. Indeed, JAK inhibitors with the clinical indication of rheumatoid arthritis (tofacitinib, baricitinib, peficitinib, upadacitinib, and flgotinib) share JAK1 as their target in common, although tofacitinib and peficitinib have dual selectivity for JAK3. However, considering that fedratinib and pacritinib have selectivity for JAK2, JAK1 possibly has a relatively more effect on the JAK-STAT signaling in rheumatoid arthritis than JAK2. Therefore, further clinical studies on the use of combination therapy of JAK inhibitors with the optimal ratio and high selectivity need to be conducted to treat rheumatoid arthritis.

Since the approval of ruxolitinib in 2011, many JAK inhibitors have been developed and launched in the global market (18–46). Although some exhibit selectivity to a certain extent, others do not. In addition, JAK inhibitors have various indications, including rheumatoid arthritis. However, a comprehensive understanding of this complexity is lacking. It remains to be determined why each JAK inhibitor has moderate selectivity and how its target, JAK, is involved in these indications. Addressing these issues could aid in the development of novel JAK inhibitors.

Recently, the FDA issued and required boxed warnings regarding the use of tofacitinib (Xeljanz), baricitinib (Olumiant), and upacitinib (Rinvoq). This action is based on the results of a clinical trial for safety, which showed a high risk of cardiovascular problems, such as heart attack and stroke, blood clots, cancer, and death (74). Although these three JAK inhibitors have clinical limitations under post-marketing surveillance, other JAK inhibitors are also being monitored in phase IV clinical trials. The current situation necessitates the development of next-generation JAK inhibitors with fewer adverse effects.

Established JAK inhibitors have been designed based on the rigid structure of the JH1 domains, most of which are crystal structures. However, as all proteins, including JAKs, are flexible, their biological actions should be understood in terms of structural flexibility. In addition, it is necessary to determine whether parts of the JH1 domain are sensitive to other domains, such as JH2, SH2, and FERM. In this context, it is necessary to understand integrated JAK biology at the molecular and cellular levels.

Members of the JAK family function as dimers in cells (15, 16). Moreover, JAKs exhibit diverse dimer combinations (4, 17). This finding implies that the quaternary structure of JAKs is associated with the recognition of specific cytokine receptors. Type I and II cytokine receptors include diverse receptors, such as type I and II interleukin receptors, interferon *α*/*β* receptors, and interferon *γ* receptors. These receptors contain peptides recognized by dimeric JAKs (4, 17). Moreover, this association between the receptor and the JAK duo occurs in the intracellular environment. Therefore, the effect of the quaternary structure of JAKs on substrate recognition is not comprehensively understood. It follows that detailed information on the entire structure of the dimeric JAKs bound to their target receptors is required.

Previous studies have illustrated the entire structures of murine JAK1 in complex with a conserved intracellular domain peptide of the cytokine receptor (15, 16). These structures provide insights into the working mechanism of the entire JAK domains, not just the JH1 domain. Caveney et al. (16) proposed a series of steps for the inhibition and activation of JAK1. During the resting period, the JH1 domain is inhibited by the JH2 domain. However, once a cytokine approaches, monomeric JAK forms a dimer with its partner, resulting in the autophosphorylation of the JH1 domain. Remarkably, this state is dynamic, and the JH1 domain recognizes intracellular terminal peptides of its receptor in a dynamic state. Finally, the receptor is phosphorylated.

If JAKs play a biological role in such a dynamic state, they may adopt diverse and specific conformations at each stage, including dimerization. This implies that novel target sites for JAK inhibitors may exist in diverse conformations. Therefore, next-generation JAK inhibitors should be developed based on a deeper understanding of the detailed dynamics of the entire JAK structure.

Recently, the importance of precision medicine has increased in the field of rheumatology. For example, a current study tried identifying predictive biomarkers for JAK inhibitors such as upadacitinib and filgotinib treatment responses of patients with rheumatoid arthritis (75). The study demonstrated that patients treated with JAK inhibitors exhibited significant differences in erythrocyte sedimentation rate, C-reactive protein, interleukin 6, soluble urokinase plasminogen activator receptor, and platelet-to-lymphocyte ratio. Personalized therapies for rheumatoid arthritis are emerging as new approaches for analyzing the personalized cytokine profiles and synovial tissue signatures of patients (76). In addition, the currently available five JAK inhibitors for rheumatoid arthritis have been evaluated as combination therapy as well as monotherapy (77). Therefore, an integrated methodology that combines personalized precision medicine with structural biology will lead to an innovative approach for treating rheumatoid arthritis.
